# The Long and Winding Road: An Overview of the Immunological Landscape of Intracranial Meningiomas

**DOI:** 10.3390/cancers14153639

**Published:** 2022-07-26

**Authors:** Giuseppe Roberto Giammalva, Lara Brunasso, Federica Paolini, Roberta Costanzo, Lapo Bonosi, Umberto Emanuele Benigno, Gianluca Ferini, Serena Sava, Cristina Colarossi, Giuseppe Emmanuele Umana, Rosa Maria Gerardi, Carmelo Lucio Sturiale, Alessio Albanese, Domenico Gerardo Iacopino, Rosario Maugeri

**Affiliations:** 1 Neurosurgical Clinic, AOUP “Paolo Giaccone”, Post Graduate Residency Program in Neurologic Surgery, Department of Biomedicine Neurosciences and Advanced Diagnostics, School of Medicine, University of Palermo, 90127 Palermo, Italy; federicapaolini94@gmail.com (F.P.); robertacostanzo3@gmail.com (R.C.); lapo.bonosi@gmail.com (L.B.); umberto.emanuele.benigno@gmail.com (U.E.B.); rosamariagerardimd@gmail.com (R.M.G.); gerardo.iacopino@gmail.com (D.G.I.); rosario.maugeri1977@gmail.com (R.M.); 2 Department of Radiation Oncology, REM Radioterapia Srl, 95125 Catania, Italy; gianluca.ferini@grupposamed.com; 3 Department of Medical Oncology, Istituto Oncologico del Mediterraneo, 95029 Viagrande, Italy; serena.sava@grupposamed.com; 4 Pathology Unit, Department of Experimental Oncology, Mediterranean Institute of Oncology, 95029 Viagrande, Italy; cristina.colarossi@grupposamed.com; 5 Department of Neurosurgery, Cannizzaro Hospital, Trauma Center, Gamma Knife Center, 95125 Catania, Italy; umana.nch@gmail.com; 6 Department of Neurosurgery, Fondazione Policlinico Universitario “A. Gemelli” IRCCS, Università Cattolica del Sacro Cuore, 00168 Rome, Italy; cropcircle.2000@virgilio.it (C.L.S.); alessio.albanese@policlinicogemelli.it (A.A.)

**Keywords:** meningioma, brain tumor immunology, infiltrating immune cells, meningioma immunology, peritumoral brain edema, meningioma immunogenetics, meningioma immunotherapy, meningioma prognosis

## Abstract

**Simple Summary:**

The tumor microenvironment represents the essential basis for characterizing oncological cells and supporting their growth. Along with genomic sequencing, the study of the tumor microenvironment represents a big step forward in oncological research and in the customization of treatments. Compared to gliomas, for which research has discovered greater results, the correlation between the microenvironment and tumor phenotype, and consequent prognostic implications, are still incompletely understood for intracranial meningioma. Recently, studies about the immunogenetic landscape of meningiomas have been promoted, and it is now clear that understanding the multifactorial pathogenesis of meningioma and its correlation with other specific signs (i.e., PTBE) could lead to the development of new targeted therapies, and significantly affect meningioma patients’ prognosis.

**Abstract:**

The role of immunotherapy is gaining ever-increasing interest in the neuro-oncological field, and this is also expanding to the management of intracranial meningioma. Meningiomas are still the most common primary adult tumor of the CNS, and even though surgery and/or radiotherapy still represent cornerstones of their treatment, recent findings strongly support the potential role of specific immune infiltrate cells, their features and genomics, for the application of personalized treatments and prognostic implications. According to the PRISMA guidelines, systematic research in the most updated platform was performed in order to provide a descriptive and complete overview about the characteristics, role and potential implications of immunology in meningioma tumors. Seventy articles were included and analyzed in the present paper. The meningioma microenvironment reveals complex immune tumor-immune cells interactions that may definitely influence tumor progression, as well as offering unexpected opportunities for treatment.

## 1. Introduction

Meningiomas are the most common primary extra-axial tumors of the central nervous system (CNS) of adults [1,2,3]. Three grades of meningiomas were described by the World Health Organization (WHO) grading system for CNS tumors until the fifth version in 2021, and several subtypes according to various criteria (cell morphology, mitosis index and levels of Ki-67) [4]. Mostly recently, due to new information, this classification was theoretically reconsidered, and new concepts like peritumoral immune cell infiltration have developed. Human tumors are generally characterized by the presence of infiltrated immune cells that have a documented role in conditioning tumor recurrence and oncological progression [5]; it is well-known that the tumor microenvironment represents the ecosystem that provides support for oncological cells to grow, and immune cells can be actors in both reducing the oncological cells’ growth and providing support in tumor feeding [6]. These observations lead to the concept of “immune surveillance”, and to the study of the peritumoral environment in order to tailor a rescue therapy when surgery cannot be considered an option for the patient [7].

Although it is widely acknowledged that the CNS is characterized by an active blood-brain barrier (BBB) that calibrates the permeability around most of the CNS blood vessels and protects the brain parenchyma, under specific stimuli and signals, circulating immune cells can also reach the CNS by migration from cerebral vessels into the perivascular space. Considering extra-assial lesions, migration to the meningioma microenvironment is even easier [6,8]. To date, the mainstay of meningioma treatment has been represented by surgery and/or radiotherapy [9], but the role of immunotherapy is gaining more attention in the neuro-oncological field, along with the recent findings mentioned above, and with medical and prognostic progress in body tumor treatment [5,10,11].

We aim to present an updated systematic review of the literature about the characteristics and role of immunology in meningioma tumor, and to provide a descriptive and complete overview about current and progressive theories, experiences, findings and potential changes in treatment and prognostic evaluation of high-recurrence meningiomas. References to meningioma grades are to the 2016 WHO classification of CNS tumors.

## 2. Materials and Methods

A systematic review of the literature was performed according to the PRISMA (Preferred Reporting Items for Systematic Reviews and Meta-Analyses) guidelines about the role of immune cells in the meningioma microenvironment and their relationship to medical therapy and tumor biology. This review was not recorded on prospective registers, thus the review protocol was not prospectively available; however, this systematic review was conducted as follows. The research was set up in May 2022, querying the PubMed and Scopus databases with the following search string: ((immunology) OR (immune cells) OR (microenvironment) OR (genomic) OR (peritumor edema) OR (peritumoral edema) or (peritumorally edema) OR (PTBE) OR (immunotherapy) OR (post-surgical treatment) OR (monoclonal antibodies)) AND ((meningioma) OR (intracranial meningioma) OR (skull base meningioma) OR (non-skull base meningioma)). Four authors (L.Br, R.C., F.P., L.Bo.) independently screened each abstract for eligibility. We included pertinent articles published until 2022. No restrictions on the type of paper were applied. Studies published in languages other than English and non-full text manuscripts were excluded. Any disagreement was solved by consensus with a third senior author (G.R.G.). Our aim was to identify recent papers focusing on the role of immunological cells in meningiomas. Our group focused on particular characteristics or cell type influencing patient prognosis and therapy, and tumor behavior

## 3. Results

**Figure 1:**
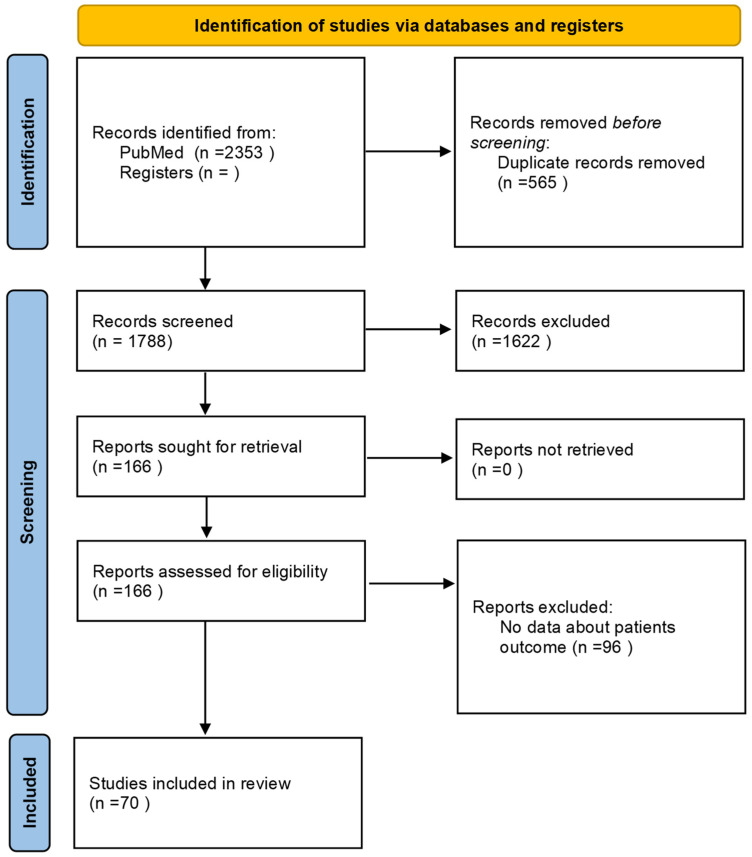
PRISMA flow diagram.

A total of 2353 studies were initially identified, which was reduced to 1788 articles after duplicate removal. After screening by title, 1427 articles were excluded. 444 papers were selected by title, and 328 by abstracts. Then, 166 studies were assessed for eligibility, and 96 studies were rejected due to the lack of reported data about patient outcome. Finally, we included in this systematic review 70 articles (Figure 1). The main results are summarized in Table 1.

## 4. Discussion

### 4.1. Immunogenetics

In the last decades, immunogenomics have earned a pivotal role in the management of meningiomas, differentiating tumor grades and, consequently, treatment responses. High-grade meningiomas, compared to low-grade ones, are related to a poor prognosis due to high-rate recurrence [20]. Meningiomas can be classified into neurofibromatosis type 2 (NF2) mutated and non-NF2 mutated.

#### 4.1.1. NF2-Mutated Meningioma

Considering the role of the NF2 gene in the regulation of leptomeningeal cell proliferation, patients affected by neurofibromatosis have an increased risk of developing meningiomatosis [21]. CREBBP, PIK3CA (R108H), PIK3R1, BRCA1, and SMARCB1 are the most represented mutations; unfortunately, none of these mutations can predict the rate of recurrence in NF2-mutated meningiomas [20,22]. Monosomy 22 and gene mutation of neurofibromin (inactivated protein) are typically found in patients with meningiomatosis, but also in both low and high-grade sporadic meningiomas [5]. Loss of chromosome 1p is another relevant mutation in WHO grade 3 rhabdoid meningiomas. Loss of 9p21, instead, is related to a poor prognosis because of the loss of the cyclin-dependent inhibitors CDKN2A and CDKN2B [5]. Otherwise, mutation of SMARCE1 or BAP1, can rarely be found in WHO grade 2 or 3 meningiomas [5]. Mutation of the NF2 locus is also linked to TERT promoter mutations, which increase with tumor grade. TERT C228T and C250T mutations are typically found in WHO grade 1 meningiomas and are associated with a higher tendency to a malignant evolution. Indeed, TERT mutations are found in histologically malignant areas, causing a wide range of heterogeneity between histologically benign and malignant regions of the same lesion [5].

#### 4.1.2. Non-NF2 Meningioma

Non-NF2 meningiomas are benign, without any chromosomal mutations. The most common genomic mutation is E17K in AKT1, a mutation activating PI3KCA and, consequently, the mTOR pathway. It is strongly related to a poor prognosis, especially in olfactory groove meningiomas. Since increased PI3KCA pathway signaling is related to aggressive behavior, these meningiomas can benefit from targeted gene therapy [20,23]. Other mutations that have recently been found are pro-apoptotic E3 ubiquitin ligase TNF receptor-associated factor 7 (TRAF7), the gene for the catalytic subunit of RNA polymerase II (POLR2A), the switch/sucrose nonfermentable (SWI-SNF) chromatin-remodeling complex gene SMARCB1, the Hedgehog pathway signaling member smoothened (SMO) and the pluripotency transcription factor Kruppel-like factor 4 (KLF4). KLF4K409Q is one of the most common somatic mutations found in benign meningiomas. The SMO (L412F) mutations are usually found in midline meningiomas, probably due to the role of the Hedgehog pathway during hemisphere separation [5,20,24]. Furthermore, patients with TRAF mutations have high levels of PD-L1, an inhibitory immune checkpoint molecule, which allows the opportunity to use immunotherapy. PD-L1 is related to T lymphocyte inhibition and exhaustion and can be overexpressed in high-grade meningiomas [25]. In addition, Karimi et al. found a higher expression of PD-L1 in anaplastic meningioma, highlighting a potential independent predictive role of this immunosuppressive factor in cancer immunity, that could be useful in routine diagnosis [26]. SMO, PI3KCA and AKT1-mTOR mutations are typically found in WHO grade 1 meningiomas with a high rate of recurrence [5,22,27]. Moreover, some mutation can lead to gene methylation patterns, influencing tumor progression. WNK2, for example, is a negative regulator of cell proliferation and its hypermethylation is related to loss of gene expression and, accordingly, to tumor aggressive behavior [20]. Osteoglycin (OGN) is located on 9q22.31 and related to bone development and vascularization. Although its role in meningioma genesis is still unclear, high OGN mRNA levels with NF2 or chromosome 22 loss have been identified [28]. In the case of a subtotal or near-total removal, an evaluation of these mutations could play a life-changing role that guarantees an optimal immunotherapeutic strategy for these kinds of patients [5].

#### 4.1.3. Familial Syndromes

There are many familial syndromes that place a patient at increased risk for meningioma development: Neurofibromatosis type 2 (autosomal dominant syndrome, mutation in NF2 suppressor gene) is associated with meningiomas in the hemispheres or in the lateral/posterior skull base; nevoid basal cell carcinoma syndrome or Gorlin syndrome (mutation of the Sonic Hedgehog pathway causing an abnormal cellular proliferation and associated to multiple basal cell carcinomas, bifid ribs, and jaw keratocysts) with meningiomas from the falx cerebri; Cowden syndrome and PTEN hamartoma tumor syndrome (PHTS—autosomal dominant disorder, mutations in phosphatase and tensin homolog (PTEN) on chromosome 10 and characterized by benign overgrowths called hamartomas as well as an increased lifetime risk of breast, thyroid, uterine cancers, and others) with anaplastic meningiomas; Werner syndrome (WRN, also called adult progeria, autosomal recessive syndrome, mutation in the WRN gene) with soft-tissue sarcomas and benign meningiomas increased incidence; BAP1 tumor predisposition syndrome (mutations of oncogene BAP1 on chromosome 3 in several neoplasms) associated with a subset of high-grade rhabdoid meningiomas; multiple endocrine neoplasia type 1 (Wermer syndrome) and Rubistein–Tabi syndrome, with only case reports and case series, recorded the association with calcified and multiple meningiomas [29].

### 4.2. Location of Meningiomas

To date, intracranial meningioma sites have been associated with specific oncogenetic molecular pathways, and many publications have focused on the correlation between the meningioma site and tumor biological behavior, documenting different features between skull base (SBMs) and non-skull base meningiomas (nSBMs), for example, calvarium meningiomas [5,54,55,56]. For instance, an analysis of 1336 patients with meningiomas revealed that location not at the skull base and age ≥ 65 years were significant risk factors for higher WHO grade (*p*-value = 0.0027 and 0.012, respectively), emphasizing the relatively benign behavior of SBMs [12].

According to the most recent literature, falcine and parasagittal meningiomas are associated with more chromosomal mutations/structural alteration than midline, lateral skull base and convexity meningiomas [5]. Yuzawa et al. reviewed three articles about 553 meningiomas whose genetic status was clarified by the NGS method and investigated the frequency of six genetic alterations (NF2, TRAF7, AKT1, KLF4, SMO, and PI1K3CA); the results showed that more than 60% of NF2-type meningiomas have a preferential localization at the level of the calvarium (convexity, parasagittal, or falcine) and are associated with more aggressive biological behavior and a higher recurrence rate [13]. These findings suggested a different genesis of meningiomas based on their location. Otherwise, 70% of TRAF7/AKT1-type meningiomas tend to localize in the anterior cranial fossa, at the medial portion of the middle cranial fossa, and at the level of the anterior convexity. SMO-type meningiomas localize, in half of the cases, at the level of the anterior cranial fossa, and in the other half, in the medial portion of the middle cranial fossa. Finally, TRAF7/KLF4-type meningiomas prefer the petroclival region, the medial portion of the posterior cranial fossa, and the lateral part of the middle cranial fossa.

Intraventricular meningiomas (IVM), although rare (about 5% of all intracranial meningiomas), also exhibit a specific tumor microenvironment with different immunogenetic characteristics depending on location. Jungwirth et al. evaluated the immunogenotype of 25 intraventricular meningiomas, and loss of chromosomes 22q and 1p frequently occurred, in 89 and 44% of cases, respectively, while NF2 mutations were found in 44% of IVMs [14]. On the other hand, in non-NF2-mutated IVMs, previously reported genetic alterations including TRAF7, AKT1, SMO, KLF4, PIK3CA, and TERT were lacking, suggesting alternative genes in the pathogenesis of non-NF2 IVMs.

It is worth noting how the cellular and particularly immunological microenvironment may play a pivotal role in defining and modulating tumor behavior [15]. Unlike in gliomas, the correlation of the microenvironment with tumor phenotype is still poorly understood in meningiomas, and research about the immunogenetic landscape of meningiomas has only recently received a boost, owing to its critical prognostic and therapeutic implications and to new sequencing techniques [16]. Through transcriptome analysis of 107 meningiomas, Zador and colleagues showed that gamma-delta T cells, monocytes, and plasma cells, with their potentially tumor-suppressant activity, are dominant in SBMs [17]; conversely, mast cells and neutrophils, with their proinflammatory and oncogenic role (related to IL-6 and IL1R2 expression), are overexpressed in convexity meningiomas. Kosugi et al. analyzed 28 meningioma tissues arising in two different locations, highlighting how an immunologically reclusive microenvironment exists in cavernous sinus (CS) meningiomas, in comparison with convexity meningiomas [18]; thus, tumor-infiltrating lymphocytes (TILs), regulatory T cells, HIF-1α, VEGF-A, and VEGFRs-1 & 2 expression, and tumor-associated macrophages (TAMs) were significantly fewer in CS meningiomas compared with convexity meningiomas. Considering that TAMs promote meningioma growth, while HIF-1α and VEGF stimulate tumor neoangiogenesis, these results confirmed the more aggressive biological behavior of convexity meningiomas, compared with SBMs [15,18].

Nevertheless, it is difficult to find a strong prognostic and therapeutic translation for these immunogenetic features, and some results still remain conflicting. For example, it has been shown that there are no statistically significant differences between SBMs and nSBMs regarding progression-free survival (PFS), tumor grading, and Ki-67 index [19].

### 4.3. Microenvironmental Features in Meningiomas: The Role of Infiltrating Immune Cells

The tumor microenvironment is a complex network of different cell types and extracellular matrix components, connecting to each other by specific signaling pathways. It is well known that each tumor has characteristic microenvironment features, required by the tumor for its growth. Thus, the microenvironment is not a static but a dynamic entity, constantly changing in time and from individual to individual, and influencing tumor behavior and the patient’s prognosis [6,32,39].

Neoplastic cells communicate with vascular endothelial cells, fibroblasts, and infiltrating immune cells with specific cytokines and chemokines. It has been demonstrated that the presence and the specific type of immune cells has an important prognostic role, and similar immune cell infiltration in different tumor microenvironments can modulate its function in an anti- or pro-tumor way. According to recent theories, neoplastic cells themselves can turn into immune cells, creating a microenvironment favorable to tumor progression [6,33].

Normally, the presence of the BBB, the absence of a resident myeloid cell population in the brain, and the lack of demonstrated lymphatic drainage create an “immunological temple” where immune cells can migrate only in certain circumstances [6]. Tumorigenesis itself induces a disruption of the BBB, thus peripheral blood immune cells are freely able to migrate through endothelial cells into the tumor microenvironment [5]. Flow cytometry (FCM) has been recently implemented for the identification and characterization of heterogeneous cell populations coexisting in meningioma tumor samples [6]. A large percentage of immune cells in the meningioma microenvironment express CD45 (macrophages, myeloid derived suppressor cells, CD8+ and CD4+ T cells, and natural killer). Meningiomas are also infiltrated by a minor percentage of both Tregs, and B cells [5].

Each immune cell subtype has specific characteristics that influence tumor biology.

#### 4.3.1. Macrophages Infiltrating Meningiomas (TAMs)

TAMs represent the largest proportion of immune cells in the meningioma microenvironment. TAMs quantity was found to be associated with tumor WHO grade and tumor size, but no influence on prognosis has yet been proved [5,57]. This cell type can modify itself by polarizing into an anti-tumor (called macrophage M1) or pro-tumor (called macrophage M2) phenotype. M1 macrophages are a strong stimulator of the immune system, and their presence is linked to a good prognosis. M2 macrophages have a different immunosuppressive effect, mostly by stimulating PD-L1 expression, and consequently promoting meningiomas’ growth and recurrence. Several studies have demonstrated the abundance of M2 macrophages and a decreased M1:M2 ratio in higher-grade meningiomas and recurrence, and it has been related to poor prognosis and a higher rate of recurrence [5,8,33]. On the other hand, M1 macrophages promote inflammation and immunostimulation by cytokine releasing, demonstrating an important tumoricidal activity, and a higher M1:M2 ratio has been associated with improved PFS. Polarization of macrophages into M1 or M2 subtypes in meningioma has been accurately studied. Hypoxia has been demonstrated as an important contributor to inducing evolution into the M2 phenotype, and is easy to find in high-grade meningiomas with necrotic areas [5,8,33,36,57].

#### 4.3.2. Microglia

Microglia and TAMs have always been identified as a single group of “tumor-associated microglia/macrophages” as a consequence of their overlapping gene expression profiles. Embryologically, microglia are derived from mesodermal tissue during early development, and are functionally related to the peripheral monocyte–macrophage cell lineage; microglia cells migrate into the brain prior to the development of the BBB, and there they are maintained through local self-renewal, and have been called the brain’s immune system [58]. TAMs, as discussed, are non-resident macrophages that invade the brain parenchyma from the periphery. It was demonstrated that microglial cells are parenchymal cells with the capacity for antigen presentation to the CNS sentinel T cells within the context of MCH class II molecules [58]. Mainly in a glioma context, it was hypothesized that microglia and macrophages, attracted by tumor cells themselves, can contribute to tumor growth through cytokine-mediated signals [5,59]. Microglial cells also have typical morphological characteristics, different from either brain macrophages or perivascular cells. Brain macrophages resemble macrophages in other organs, with their typical round or ovoid cell shape and lack of ramified cell processes; perivascular cells are relatively large and spindle-shaped, and situated near to CNS blood vessels [58]. Thus, microglia and TAMs share immunological functions including phagocytosis and antigen presentation, but they are ontogenetically distinct and it has been found out that each presents unique transcriptomes and epigenetic signatures, driving differential functions within the tumor microenvironment. It has been widely reported that a high myeloid load correlates with poor patient survival in a range of tumors [60]. Some studies have investigated the role of a microglial/macrophagic response in meningiomas lacking an intervening pial–glial basement membrane, in contrast to tumors that are separated from the brain by a basement membrane; the intactness of the basal membrane of invasive meningiomas correlated with malignancy grades, and invasive meningiomas are expected to break the membrane [61]. Nonetheless, the independent prognostic capacity of microglia is yet to be fully investigated.

#### 4.3.3. Myeloid Derived Suppressor Cells (MDSCs)

MDSCs represent a heterogeneous group of immature myeloid cells, identified through a characteristic immune-phenotype similar to marrow stem cells. This immune cell type has been isolated, both in fresh meningioma samples and in peripheral blood of the same patients [6,33]. While in peripheral blood no activity was found, in tumor samples they showed a strong immunosuppressive activity, representing an important way of a tumor escaping. They play a direct role in tumorigenesis by promoting tumor vascularization and enhancing PD-L1 and NF2 expression [8]. Recent work suggests that MDSCs induce an impaired immune activity by blocking CD8+ T cell and NK cells activation, M1 polarization, tumor antigen presentation to DCs, and inducing M2 switch [5,6,8,33]. MDSCs are mostly represented in grade 2 or 3 meningiomas [5,6].

#### 4.3.4. Mast Cells

Mast cells (MCs) play an important role in cancer promoting, by producing a large amount of metalloproteinases and thus contributing to the tumor diffusion process, and by secreting prestored mediators such as corticotropin-releasing hormone (CRH), neurotensin (NT), substance P (SP), tryptase, vascular endothelial growth factor (VEGF), tumor necrosis factor (TNF), prostaglandins, and leukotrienes, some of which are known to disrupt the integrity of the BBB, stimulating peritumoral brain edema (PTBE) formation [9,41,43]. Reszec and colleagues analyzed 70 cases of meningiomas and documented the presence of MCs within the tumor and, remarkably, in peri-vascular areas; their study showed the correlation, not only between MCs infiltrate and PTBE, but also between MCs and recurrence rate or a bad prognosis of meningiomas [40]. Furthermore, these results were confirmed by the discovery that secretory meningiomas, a particular subtype often associated with extensive PTBE, express a high concentration of MCs compared with non-secretory meningiomas [42]. However, their role in meningioma progression is still debated. In fact, the microenvironment of both low grade and high-grade meningiomas contains MCs. Whereas in low-grade meningiomas MCs are located next to blood vessels, in high grade lesions their presence is diffuse in all of the tumor section [9,40,43].

#### 4.3.5. Treg

High grade meningiomas show higher percentages of Tregs compared to low grade lesions [5,6,17]. As we know, tumor-infiltrating myeloid cells in meningiomas are mainly CD3+ T cells, both CD8 and CD4, and natural killer cells, and Tregs to a lesser extent. In the meningioma microenvironment, their role is still debated [5,44]; their percentage seems to be related to meningioma grading. Interestingly a recent study showed higher infiltrates of Tregs and lower infiltrates of CD4 and CD8 T-cells, compared to low grade lesions [44]. In particular, 90% of grade III meningiomas exhibited Treg infiltration compared to 33% of grade I and 28% of grade Il. These data support their immunosuppressive role, contributing to tumor progression. In fact, Treg are inhibitory T cells, and their negative impact on survival has been demonstrated in different tumor types. In meningiomas, Treg infiltration has not been demonstrated as an independent prognostic factor influencing grading and consequent outcome [44].

#### 4.3.6. Natural Killers (NK)

NK are a lymphocyte subtype with a proved antitumoral effect via ADCC killing, and their effect is blocked by the PD-1/PD-L1 system. Recent studies have demonstrated that Avelumab, a PD-L1 blocking antibody, can improve ADCC killing in brain tumors where the tumor microenvironment is rich in NKs [38].

### 4.4. Peritumoral Brain Edema (PTBE)

PTBE is frequently reported in imaging for intracranial meningiomas, ranging in incidence from 38% to 67%. It seems to be associated with higher mortality and morbidity, potentially influencing the surgical strategy in tumor removal. [45] The pathogenesis of PTBE is still unclear, and four pathogenetic hypotheses are particularly popular: (1) “the brain parenchyma compression theory” directly related to the tumor size, where PTBE is the result of ischemic phenomena deriving from direct compression of the surrounding parenchyma and consequent cytotoxic edema [46,62]. (2) “the secretory–excretory theory”, referring to the significant correlation between PTBE volume and tumor histotype (particularly the secretory subtype) which is found to be associated with more pronounced expression of cytokeratin (CK) and carcinoembryonic antigen (CEA), both closely related to cytotoxic edema [47]. (3) “the venous compression theory”, where increased intra-tumor venous pressure could lead to tumor congestion, increase in vasogenic substances and cerebral–pial capillary permeability, and PTBE formation and expansion through vasogenic edema production [48]. (4) “the hydrodynamic theory”, according to the idea that tumor stasis occurs not only because of the compression of an adjacent cortical vein, but mostly from poor development of the tumor’s drainage system; when tumor blood supply becomes insufficient, meningiomas secrete angiogenic factors (such as VEGF-A, Endothelin-1, Caveolin-1) resulting in immature permeable neovessels, leakage of plasma proteins, and PTBE development in the surrounding brain parenchyma [45,49,63].

For a long time, it was assumed that PTBE around meningiomas could be explained by a physical mechanism alone, but, to date, it seems well established that alterations in certain molecular pathways, release of growth factors, and tumor microenvironment composition may play a crucial role in its pathogenesis. Many studies have focused on the role of VEGF, a regulator of angiogenesis and vascular permeability modulated at the transcriptional and post-transcriptional level by hypoxia, and its pathway [64,65,66,67]. Some authors have investigated the role of the VEGF-A pathway in the pathogenesis of PTBE in meningiomas, highlighting how an increase in VEGF-A expression was associated with increased peritumoral capillary length and PTBE [50]; this association showed, in some cases, low statistical significance [68,69,70]. Iwado’s group, in a retrospective study of 60 grade I meningiomas, showed that VEGF levels were directly proportional to the development and extent of PTBE (*p*-value = 0.0397); interestingly, they also analyzed the relationship between metalloprotease-9 (MMP-9) and PTBE, and together VEGF and MMP-9 correlated with PTBE presence (*p*-value = 0.062, not statistically significant), potentially inducing the disruption of the arachnoid membrane and formation of the pial blood supply [71]. The role of hypoxia-inducible factor-1 (HIF-1), a transcription factor implicated in tumorigenesis and tumor neoangiogenesis, in PTBE formation was also investigated, and it has been shown how, under hypoxic conditions, overexpression of HIF-1 leads to BBB disruption and PTBE formation in meningiomas [51].

The method by which the extracellular matrix actively participates in edema formation in meningiomas, supporting the multifactorial hypothesis underlying PTBE, was interestingly reported by Kilic et al. [52]; they demonstrated that a correlation between tenascin (a matrix protein involved in several biological process such as embryogenesis, wound healing, reactive astrocytosis, and in some pathological processes, including tumor-associated angiogenesis) expression and peritumoral edema in meningiomas exists. Many other publications have considered other markers and molecular pathways directly implicated in the genesis of PTBE, including the role played by aquaporins [53,72,73], adhesion molecules [74], cytokines [75] and other factors [76].

### 4.5. Immunotherapy

Scientific evidence supports the theory that maximal safe surgical resection of the tumor associated with the resection of as much as possible of the dural margins significantly decreases the risk of tumor recurrence; thus, surgical treatment still remains the mainstay of treatment for meningiomas. The anatomical location of the meningioma represents one of the unmodifiable prognostic factors influencing the ability to resect the lesion entirely with wide dural margins [5]. For recurrences, additional lines of treatment are available after surgery, including adjuvant radiotherapy such as fractionated stereotactic radiation (FSR) or stereotactic radiosurgery (SRS) [5]. Even in cases of complete surgical resection, a subset of meningiomas exhibits aggressive behavior and recurs. Recent data show that grade I meningioma has a low 5-year recurrence rate after surgery, ranging from 0–22.5%; in contrast, the 5-year recurrence in atypical and anaplastic meningioma is reported to be 50% and 80%, respectively [5,31,77].

Recurrent meningiomas represent a difficult challenge for both patients and clinicians [78]. The role of chemotherapy (alone or as an adjuvant treatment after surgery) and radiotherapy is limited due to the lack of evidence of its beneficial aspects. Specific chemotherapeutic agents (doxorubicin, irinotecan, vincristine, and temozolomide) have been evaluated and found not to be effective in improving PSF for HGMs [30], in contrast to other solid tumors. The importance of deregulated cell signaling pathways as drivers of neoplastic transformation is increasingly gaining attention, and several studies have suggested a critical role of VEGF in meningioma pathogenesis, as its expression correlates with tumor grade, PTBE, and necrosis [5,31]. High expression of VEGF was also considered a predictive factor for higher risk recurrence [5]. Thus, antiangiogenic agents have been evaluated and found to have suboptimal results [5,31]. Receptor tyrosine kinase inhibitors (gefitinib, erlotinib, and imatinib) that target platelet derived and epidermal growth factor receptors (PDGF and EGF, respectively), and treatment targets on genetic alterations (mutations in AKT1, PIK3CA, SMO and NF2) have also been tested in clinical trials [5,32,33]. Scientific evidence supports the use of immunotherapy as an effective target treatment strategy for several solid tumors, such as lung cancer and melanoma, showing improved survival rates [79]. Immunotherapy has already been considered for treating other brain tumors, particularly glioblastoma (GBM), although the poor results were not satisfactory [80]. Therefore, encouraging results in the use of immunotherapy make it a potentially attractive therapeutic alternative for HGMs, as a potential solution to overcome some of the limitations of conventional treatments (surgery, radiotherapy and chemotherapy). The objective of target-therapy is to meet the target, to leverage on it and to benefit from it, and the target is the immunosuppressive microenvironment in meningiomas, which may contribute to tumor progression, and the host immune system that may be used against the tumor itself. Recently, research has focused on utilizing monoclonal antibodies targeting PD1/PD-L1. PD-L1 is expressed on the surface of tumor cells, and it is used to evade the immune system because it inhibits T-cell activation by binding to the PD-1 surface receptor on T- and B-cells. The expression of PD-L1 has been shown to impact on tumor grade in meningiomas and it is considered one of the major mechanisms used by meningiomas to evade the host immune system [34]. Moreover, patients with grade III meningiomas have also shown increased MDSCs that overexpress PD-L1, corroborating the hypothesis of systemic immunosuppression in meningioma patients [5]. Considering those patients who have received prior radiation therapy and who have also shown significantly higher expression of PD-L1, PD1 blockade therapy is considered a potential, viable and successful treatment strategy to treat patients with high grade and also recurrent meningiomas. There are still many open questions, because it is not yet clear whether level of expression will correlate with treatment response. Lesser-known immunomodulatory proteins, such as PD-L2, B7-H3, CTLA-4, and NY-ESO-1, were also identified as highly expressed molecules in meningiomas [8,35,36]. The expression of PD-L2, which is a further receptor for PD-1, and B7-H3 was significantly higher in patients carrying genetic mutations in the PI3K/AKT/mTOR pathway, and CTLA-4 in those with mutations in PIK3CA or SMO. PD-L2 was found to be expressed at higher levels throughout all meningioma grades, compared to PD-L1, and it has been presumed that it could play a role as a predictor for immunotherapy response [8]. The exact mechanism of B7-H3 interactions is yet not known, but its blockade (antibodies anti-B7H3) is showing consistent results in reducing tumor growth in early clinical trials for other tumors, including gliomas, suggesting it as a potential and promising meningioma therapeutic target [35]. There are currently clinical trials enrolling patients to receive PD-1 blockade immunotherapy (Nivolumab, Avelumab, Pembrolizumab), comparing them to anti-CTLA-4 antibody (Ipilimumab) [8]. NY-ESO-1 has been demonstrated to contribute to both humoral and cellular immune responses in other tumors aside from meningioma, with a correlation reported between tumor grade, patient outcomes and NY-ESO-1 levels of expression [37]. There are several prospective and clinical trials testing NY-ESO-1 based immunotherapies. Other therapeutic strategies apart from checkpoint inhibitors are promising, including chimeric antigen receptor T cells (CAR-T cells), showing potential effects on levels of immunosuppression in the tumor immune microenvironment. Adequate dosing and differing targets are being explored to draw conclusions on the potential of CAR-T therapy, especially for refractory meningiomas [5]. Allogenic NK lymphocyte infusions are showing beneficial results in other solid tumors, and it is reasonable to study this alternative for meningiomas [38].

## 5. Conclusions

Meningiomas are not limited by the BBB, and they are known to show a complex immune microenvironment where immunomodulatory protein expression, genetic alterations, immune cells infiltrations and tumor-immune cells interactions form a dynamic prognostic interplay. Even though, compared to glioma, changes and applications of meningiomas are slower, scientific research is journeying towards countless new frontiers in the meningioma world. The immune microenvironment in meningiomas is well-established as a key factor in the prognostic evaluation, and this is leading to defining optimal and personalized treatment where the oncological challenge is harder to win, or intended as preoperative, and surgical treatment may be not sufficient. Significant findings have been achieved, but more larger and comparative studies are needed to determine conclusive results and discover a better approach to the treatment of meningioma patients.

## Figures and Tables

**Table 1:** Result of systematic review and summary of main data from selected articles.

	**Location**
Cornelius et al., 2013 [12]	Location not at the skull base and age ≥65 years are significant risk factors for higher WHO grade
Garzon-Muvdi et al., 2020 [5]	Falcine and parasagittal meningiomas associated to chromosomal mutations/structural alteration
Yuzawa et al., 2016 [13] Jungwirth et al., 2019 [14]	Higher frequency of NF2, TRAF7, AKT1, KLF4, SMO, and PI1K3CA mutations Loss of chromosomes 22q (89%) and 1p (44%) and NF2 mutation (44%) in IVMs
Yeung et al., 2021 [15] Terabe et al., 2021 [16]	Immunological microenvironment defines and modulates tumor behavior with prognostic and therapeutic implications
Zador et al., 2020 [17] Kosugi et al., 2019 [18]	Gamma-delta T cells, monocytes, and plasma cells dominant in SBMs Mast cells and neutrophils overexpressed in convexity meningiomas TILs, Treg, HIF-1α, VEGF-A, VEGFRs-1 & 2, TAMs significantly lower in CS
Savardekar et al., 2018 [19]	No differences in PFS, tumor grading and Ki-67 index between SBMs and nSBMs
	**Immunogenetics**
Al-Rashed, et al., 2020 [20]	Increasing meningioma grade associated with increased VEGF, Ki67, TOP2, PD-1, and PDGFRB Cytostatic mTOR inhibitors promising in controlling tumor growth Inhibition of EZH2 potentially improve outcomes
Goutagny et al., 2010 [21]	NF2 loss associated with chromosome instability Most grade I meningiomas do not progress to a higher grade and are characterized by very few chromosome alterations, mainly isolated 22q loss
Brastianos et al., 2013 [22]	A subset of meningiomas lacking NF2 alterations harbored oncogenic mutations in AKT1 (E17K) and SMO (W535L) These mutations were present in therapeutically challenging SBMs and higher-grade tumors
Strickland et al., 2016 [23]	Mutation rates at high frequency for SMO (11%) and AKT1 (19%) in both WHO Grade I and Grade II anterior skull base meningiomas Genotyping of SMO and AKT1 is likely to be high yield in anterior skull base meningiomas with available surgical tissue
Bi et al., 2016 [24]	Recurrent somatic mutations in NF2, TRAF7, KLF4, AKT1, SMO, and PIK3CA are collectively present in ~80% of sporadic meningiomas The recent identification of AKT1, SMO, and PIK3CA mutations opens the door for targeted pharmacotherapeutics in ~20% of grade I meningiomas.
Hao et al., 2019 [25]	KLF4- and TRAF7-mutated tumors were predominantly secretory skull base meningiomas SMO-mutated tumors exhibited higher calcification, and half of these tumors were observed in the brain midline TRAF7 mutations could play a key role in skull base meningiomas by regulating the expression of inhibitory immune checkpoints and suppressing immune responses
Karimi et al., 2020 [26]	A significantly positive relationship between higher PD-L1 expression and grading is shown PD-L1 expression levels represent an independent prognostic factor to predict tumor recurrence Hypoxia is one of the potential regulatory mechanisms for PD-L1 expression in meningioma
Williams et al., 2019 [27]	A large subset of posterior fossa meningiomas (foramen magnum) harbor AKT1 E17K mutations and are therefore potentially amenable to targeted medical therapy In contrast to AKT1 mutations, SMO or PIK3CA mutations were absent in the posterior fossa
Mei et al., 2017 [28]	OGN contribute to meningioma cell growth through interaction with NF2, AKT, and mTOR OGN downregulate NF2, the canonical tumor suppressor altered in approximately half of meningiomas AKT inhibition reduces OGN protein levels in meningioma cells, with a concomitant increase in cell death
Kerr et al., 2018 [29]	Combining histology, genetics, epigenetics, and clinical findings will provide the best system for classification Increased risk of meningioma: NF2, nevoid basal cell carcinoma syndrome, multiple endocrine neoplasia 1 (MEN1), Cowden syndrome, Werner syndrome, BAP1 tumor predisposition syndrome, Rubinstein-Taybi syndrome, and familial meningiomatosis caused by germline mutations in the SMARCB1 and SMARCE1 genes
	**Immunotherapy**
Garzon-Muvdi et al., 2020 [5]	Role of VEGF in meningioma pathogenesis, and its expression correlates with tumor grade, peritumoral edema, and necrosis Receptor tyrosine kinase inhibitors that target PDGF and EFG in clinical trial Grade III meningiomas showed increased MDSCs that overexpress PD-L1, corroborating the hypothesis of the systemic immunosuppression Utilization of monoclonal antibodies targeting PD1/PDL1 CAR-T cells show potential effects on immunosuppression in tumor microenvironment Promising clinical trials testing CAR-T therapy, especially for refractory meningiomas
Wen et al., 2010 [30]	Specific chemotherapeutic agents (doxorubicin, irinotecan, vincristine, and temozolomide) not effective in improving PSF for HGMs
Scerrati et al., 2020 [31]	High expression of VEGF is a predictor factor for higher risk recurrence Antiangiogenic agents evaluated with suboptimal results
Garzon-Muvdi et al., 2020 [5] Domingues et al., 2012 [32] Pinton et al., 2018 [33]	Treatment targets on mutations in AK1, PIK3CA, SMO, and NF2 in clinical trials
Garzon-Muvdi et al., 2020 [5] Arasanz et al., 2017 [34]	The use of immunotherapy could become an alternative for HGMs over the limits of the conventional therapy The target is the immunosuppressive microenvironment PD-L1 is expressed on the surface of tumor cells, and it inhibits T-cell activation by binding to the PD-1 receptor on T- and B-cells. It is one of the major mechanisms used by meningiomas to evade the host immune system
Proctor et al., 2019 [8] Flem-Karlsen et al., 2018 [35] Han et al., 2016 [36]	PD-L2 (receptor for PD-1), B7-H3, CTLA-4, and NY-ESO-1 were highly in meningiomas, and in patients who carry genetic mutations in PI3K/AKT/mTOR pathway PD-L2 is overexpressed throughout all meningioma grades, and it has been presumed to play a role as a predictor for immunotherapy response CTLA-4 were highly in patients with carry genetic mutations in PIK3CA or SMO Clinical trials for the use of PD-1 blockade immunotherapy (Nivolumab, Avelumab, Pembrolizumab), comparing with anti-CTLA4 antibody (Ipilimumab)
Thomas et al., 2018 [37]	NY-ESO-1 contributes in both humoral and cellular immune responses apart from tumor grade Promising clinical trials testing NY-ESO-1 base immunotherapies
Giles et al., 2019 [38]	Allogenic NK lymphocytes considered a reasonable alternative for meningiomas
	**Immune cells infiltrate**
Giles et al., 2019 [38]	NK lymphocyte subtype with antitumoral effect by ADCC killing Their effect is blocked by PD-1/PD-L1 system
Garzon-Muvdi et al., 2020 [5] Proctor et al., 2019 [8] Pinton et al., 2018 [33] Proctor et al., 2019 [8] Han et al., 2016 [36] Rossi et al., 1988 [39]	Higher B7- H3, and PD-L2 l in patients with PI3K/AKT/mTOR pathway mutations Higher CTLA-4 in PIK3CA or SMO mutations. Higher expression of PD-L2 compared to PD-L1 throughout all meningioma grades NY-ESO-1 provokes humoral and cellular immune responses. TAMs represent the largest part of immune infiltrate of meningiomas They are able to polarize into M1 phenotype (anti-tumor, stimulator of immune system, better prognosis) or M2 (pro-tumor, immunosuppressive effect by stimulating PD-L1 expression, promoting meningioma growth and recurrence) Decreased M1:M2 ratio in higher meningiomas with higher recurrence rate, and higher M1:M2 ratio associated with improved PFS
Garzon-Muvdi et al., 2020 [5] Pinton et al., 2018 [33] Proctor et al., 2019 [8] Domingues et al., 2016 [6]	MDSCs, a heterogeneous group of immature myeloid cells, with immunosuppressive activity and most represented in grade 2 or 3 meningiomas Role in promoting vascularization, enhancing PD-L1 and NF2 expression, and tumorigenesis and tumor escaping Newly discovered role in reducing immune activity by blocking CD8+ T cell and NK activation, M1 polarization, tumor antigen presentation to DCs, and inducing M2 switch
Polyzoidis et al., 2015 [9] Reszec et al., 2012 [40] Domingues et al., 2012 [32] Theoharides et al., 2012 [41] Tirakotai et al., 2006 [42] Schober et al., 1988 [43]	MCs important role in cancer promoting Role in producing metalloproteinases and secreting CRH, NT, substance P, tryptase, VEGF, TNF, prostaglandins, leukotrienes, and thus contributing to tumor diffusion and to disruption the integrity of the BBB and stimulating PTBE formation MCs association to recurrence rate and bad prognosis of meningiomas is still debated, and microenvironment of both low and high grade meningiomas contains MCs
Zador et al., 2020 [17] Garzon-Muvdi et al., 2020 [5] Li et al., 2019 [44] Domingues et al., 2016 [6]	Treg are inhinitory T cells Their percentage is higher in higher grade meningiomas supporting their immunosuppressive potentiality Their negative impact on survival has been demonstrated in different tumor types Their effective role in meningiomas is still debated, and Treg infiltration has not been yet demonstrated as independent prognostic factor
	**PTBE**
Berhouma et al., 2019 [45]	Incidence ranges from 38% to 67% and is associated with higher mortality and morbidity
Gilbert et al., 1983 [46]	PTBE is related to ischemic-compressive phenomena related to tumor size
Regelsberger et al., 2009 [47]	Some meningioma histotypes correlate with a more extensive PTBE Secretory hystotype is associated with higher CK and CEA expression
Bitzer et al., 1997 [48]	Increased intratumoral venous pressure leads to tumor congestion and expansion of the PTBE
Tanaka et al., 2006 [49]	Hydrodynamic mechanism results in altered intratumoral rheology, venous congestion and release of vasogenic substances
Nassehi et al., 2013 [50] Reszec et al., 2013 [51]	Alterations to the VEGF pathway appear to be implicated in the pathogenesis of PTBE Increase in MMP-9 and HIF-1 correlates with a higher PTBE
Kilic et al., 2002 [52] Gawlitza et al., 2017 [53] Polyzoidis et al., 2015 [9]	Other molecular alterations underlying PTBE appear to involve ECM protein or membrane ion channel protein Mast cells promote BBB destruction by increasing PTBE

IVMs: intraventricular meningiomas; TILs: tumor-infiltrating lymphocytes; Treg: regulatory T cells; TAMs: tumor-associated macrophages; PFS: progression free survival; SMBs: skull base meningiomas; nSMBs: non-skull base meningiomas; PTBE: peritumoral brain edema; MDSCs: myeloid derived suppressor cells.
